# Supplementary material for: Deep learning-based age estimation from chest X-rays indicates cardiovascular prognosis
Source: Commun Med (Lond). 2022 Dec 9;2:159. doi: 10.1038/s43856-022-00220-6 (PMC9734197; doi:10.1038/s43856-022-00220-6)
Supplement: Supplementary file 1 — Supplementary Information [file 43856_2022_220_MOESM1_ESM.pdf]

Supplementary Information

Supplementary Tables

Supplementary Table 1. Estimation accuracy of age model in the validation dataset using different deep learning architectures.

Architecture	MSE	RMSE	R	MAE
ResNet18	35.0	5.91	0.936	4.54
ResNet34	32.7	5.72	0.940	4.42
ResNet50	30.0	5.48	0.946	4.20
ResNet101	29.8	5.46	0.946	4.20
ResNet152	32.8	5.73	0.942	4.22
DenseNet121	30.7	5.54	0.945	4.25
DenseNet161	30.3	5.50	0.945	4.22
DenseNet169	33.4	5.78	0.939	4.25
DenseNet201	27.7	5.26	0.949	4.04
Inception v4	31.0	5.57	0.945	4.32
**SENet154**	**27.3**	5.23	0.952	4.08

The model with the smallest mean squared loss in the validation dataset was selected as the final model. MSE, mean squared error; RMSE, root mean squared error; R, Pearson's r between the chronological and estimated age; MAE, mean absolute error.

Supplementary Table 2. Data split in 5-fold cross validation analysis

	Dataset	Number of CXR	Number of Patients
CV1	Training	78,831	21,561
	Validation	11,028	3,080
	Test	22,245	6,161
CV2	Training	77,569	21,561
	Validation	11,294	3,080
	Test	23,241	6,161
CV3	Training	78,927	21,561
	Validation	10,898	3,080
	Test	22,279	6,161
CV4	Training	78,872	21,561
	Validation	11,280	3,080
	Test	21,952	6,161
CV5	Training	78,566	21,561
	Validation	11,146	3,080
	Test	22,392	6,161

Number of CXR images and patients included in each split in the 5-fold cross validation analysis. The NIH chest X-ray dataset was divided into training, validation, and test datasets in a ratio of 7:1:2 of patients, with no overlap of patients.

Supplementary Table 3. Five-fold cross validation result for age estimation model

Dataset	Test			JSRT		
	R	MAE	MSE	R	MAE	MSE
cv1	0.951	4.05	27.82	0.915	5.25	45.20
cv2	0.949	4.20	29.10	0.912	4.96	41.84
cv3	0.954	4.00	26.47	0.920	4.83	38.58
cv4	0.949	4.03	27.30	0.922	4.61	37.37
cv5	0.949	4.17	29.08	0.919	5.63	51.67
CV result	0.950	4.09	27.96	0.917	5.05	42.93
Original split	0.962	3.67	21.25	0.916	4.95	41.92

Estimation performance on the test and JSRT datasets in each split; the averaged estimation performance is shown. R, Pearson's r between the chronological and estimated age; MAE, mean absolute error; MSE, mean squared error; CV, cross validation

Supplementary Table 4. Estimation performance of DNN model trained using only "No finding" data

Dataset	Test dataset			JSRT dataset		
	Model trained with All data	Model trained with "No finding" only	P value	Model trained with All data	Model trained with "No finding" only	P value
R	0.962	0.951	< 0.0001	0.916	0.908	0.135
MAE	3.666	3.969	0.0013	4.953	5.214	0.059
ICC	0.957	0.945	0.0005	0.878	0.866	0.0385

Estimation performance of DNN model trained using only "No finding" CXR images and the model trained with all images are given. The P value was derived using the 20,000 bootstrap replications method. R, Pearson's r between the chronological and estimated age; MAE, mean absolute error; ICC, intraclass correlation coefficient; DNN, deep neural network.

Supplementary Table 5. Age estimation performance in different patient age groups.

Dataset	Subset	R			MAE			ICC		
		Estimate	C.I.		Estimate	C.I.		Estimate	C.I.	
			lower 95%	upper 95%		lower 95%	upper 95%		lower 95%	upper 95%
Test	≥ 65 y/o	0.718	0.624	0.792	4.47	3.91	5.02	0.472	0.353	0.576
	18 y/o <, < 65 y/o	0.921	0.906	0.933	3.45	3.21	3.69	0.915	0.902	0.927
	≤ 18 y/o	0.954	0.870	0.984	3.34	2.57	4.12	0.793	0.572	0.907
JSRT	≥ 65 y/o	0.500	0.322	0.644	6.78	5.64	7.93	0.130	-0.048	0.300
	< 65 y/o	0.904	0.871	0.929	3.96	3.50	4.20	0.893	0.863	0.917

Different metrics of age estimation performance in older (≥ 65 years old), middle-aged (18 y/o <, < 65 y/o), and younger (≤ 18 years old) patients in the test and JSRT datasets. There were no participants under the age of 18 in the JSRT dataset (Supplementary Fig.1). R, Pearson's r between the chronological and estimated age; MAE, mean absolute error; ICC, intraclass correlation coefficient.

Supplementary Table 6. Estimation performance of a single DNN model and an ensemble model.

Dataset	Test dataset			JSRT dataset		
	SENet154 model	Ensemble prediction model	P value	SENet154 model	Ensemble prediction model	P value
R	0.962	0.960	1	0.916	0.912	1
MAE	3.666	3.711	0.558	4.953	4.709	0.075
ICC	0.957	0.955	1	0.878	0.889	0.053

Estimation performance of SENet154-based single DNN model and the ensemble estimation model of 11 different DNN architectures. The P value was derived using 20,000 bootstrap replications. R, Pearson's r between the chronological and estimated age; MAE, mean absolute error; ICC, intraclass correlation coefficient.

Supplementary Table 7. Summary of human physician estimation of age

		R			MAE			ICC		
		Estimate	C.I.		Estimate	C.I.		Estimate	C.I.	
			lower 95%	upper 95%		lower 95%	upper 95%		lower 95%	upper 95%
Individual estimation	Physician1	0.483	0.380	0.573	14.33	13.03	15.63	0.384	0.290	0.470
	Physician2	0.204	0.081	0.321	12.33	11.05	13.61	0.200	0.097	0.298
	Physician3	0.440	0.333	0.536	17.84	16.40	19.29	0.118	0.014	0.221
	Physician4	0.392	0.280	0.493	12.43	11.19	13.67	0.392	0.300	0.478
	Radiologist1	0.587	0.499	0.664	11.90	10.78	13.02	0.567	0.491	0.634
	Radiologist2	0.542	0.447	0.625	14.65	13.22	16.08	0.376	0.282	0.462
	Radiologist3	0.716	0.649	0.772	14.33	13.16	15.51	0.388	0.295	0.474
Mean estimation performance	All doctors	0.481	0.331	0.630	13.97	12.08	15.86	0.346	0.211	0.481
	Physicians	0.380	0.184	0.575	14.23	10.13	18.34	0.274	0.057	0.490
	Radiologists	0.615	0.391	0.840	13.63	9.89	17.36	0.443	0.178	0.709
Ensemble prediction	All doctors	0.698	0.627	0.757	10.06	9.17	10.94	0.549	0.471	0.618
	Physicians	0.550	0.456	0.631	10.70	9.72	11.69	0.444	0.355	0.524
	Radiologists	0.712	0.644	0.769	10.99	9.95	12.02	0.559	0.482	0.627
DL model	DL_model	0.916	0.893	0.934	4.95	4.43	5.48	0.878	0.852	0.900

Age estimation performance of human physicians and radiologists in the JSRT dataset; the predictive performances of four physicians and three radiologists are given. The mean estimation performance is the average of the doctors' metrics.

Ensemble prediction is a metric of ensemble prediction by multiple doctors (see the Methods section). R, Pearson's r between the chronological and estimated age; MAE, mean absolute error; ICC, intraclass correlation coefficient; DL_model, deep learning model.

Supplementary Table 8. Characteristics of heart failure patients

Clinical measurements (unit)	Heart failure patients (n=1562)
Age (years old)	78 [69, 84]
The number of males (%)	920 (58.9)
Height (cm)	159 [151.5, 166.5]
Weight (kg)	58.3 [50, 67.8]
BMI (kg/m^2)	23.1 [20.7, 25.8]
Etiology of heart disease	
ischemic	424 (27.1)
valvular	537 (34.4)
other	601 (38.5)
Hypertension	945 (60.5)
Diabetes mellitus	446 (28.6)
Dyslipidemia	625 (40.0)
Smoking history	760 (48.7)
Atrial fibrillation/atrial flutter	974 (62.4)
HOT	40 (2.6)
Implantable device	
None	1359 (87.0)
PM	127 (8.1)
ICD	53 (3.4)
CRT	23 (1.5)
Systolic BP (mmHg)	137 [119, 157]
Diastolic BP (mmHg)	79 [66, 95]
Heart rate (min)	89 [71, 110]
SpO_2 (%)	95 [92, 98]
Clinical scenario	
CS1	728 (46.6)
CS2	719 (46.0)
CS3	115 (7.4)
Laboratory measurements	
Hb (g/dl)	12.0 [10.5, 13.6]
Hct (%)	37.0 [32.6, 42.0]
BUN (mg/dl)	21.2 [16.5, 29.4]
Na (mEq/L)	140 [137, 142]
K (mEq/L)	4.4 [4.0, 4.7]
T-Bil (mg/dl)	1.0 [0.7, 1.4]
AST (U/L)	35 [26, 52]
ALT (U/L)	23 [15, 41]
ALP (U/L)	292.38 (216.32)
Albumin (U/L)	3.7 [3.3, 3.9]
UA (U/L)	6.4 [5.3, 7.8]
NT-proBNP (pg/ml)	3777 [1952, 7653]
CRP (mg/dl)	0.43 [0.14, 1.40]
Cre (mg/dl)	1.00 [0.79, 1.33]
eGFR (ml·min^{-1}·1.73 m^{-2})	51.2 [36.47, 64.07]
WBC	6400 [5100, 8200]
Lymph (%)	20.80 [14.70, 28]
BS (mg/dl)	124 [105, 157.5]
TSH (µIU/ml)	2.44 [1.51, 4.21]
HbA1c (%)	5.9 [5.5, 6.4]
TC (mg/dl)	159 [135.75, 185]
Echocardiography parameters	
LVDd (mm)	51 [44, 58]
LVDs (mm)	38 [31, 49]
LVEF (%)	47 [31, 58]
LAD (mm)	45 [40, 51]
TRPG (mmHg)	28 [22, 37]

Characteristics of patients with HF. Continuous variables are presented as medians [interquartile ranges], except for ALP. ALP is expressed as mean (standard deviation) as it is normally distributed; the Shapiro–Wilk test was used to check for it. Categorical variables are presented as n (%).

BMI, body mass index; HOT, home oxygen therapy; PM, pacemaker; ICD, implantable cardioverter defibrillator; CRT, cardiac resynchronization therapy; Hb, hemoglobin; BUN, blood urea nitrogen; T-Bil, total bilirubin; AST, aspartate aminotransferase; ALT, alanine aminotransferase; ALP, alkaline phosphatase; UA, urinary acid; CRP, C-reactive protein; Cre, creatinine; eGFR, estimated glomerular filtration rate; WBC, white blood cell count; Lymph, lymphocytes; BS, blood glucose; TSH, thyroid stimulating hormone; HbA1c, hemoglobin A1C; TC, total cholesterol; LVDd, left ventricular end-diastolic diameter; LVDs, left ventricular end-systolic diameter; LVEF, left ventricular ejection fraction; LAD, left atrial dimension; TRPG, tricuspid regurgitation peak gradient.

Supplementary Table 9. Cox proportional hazards model for primary endpoint in heart failure patients.

Variable (unit)	Coefficient	HR	Confidence interval		z	P value
			lower 95%	upper 95%		
Age (years)	0.039	1.040	1.032	1.048	9.566	1.11×10^{-21}
Sex (Male)	-0.153	0.858	0.732	1.006	-1.889	5.89×10^{-2}
BMI (kg/m^2)	-0.053	0.949	0.929	0.969	-4.900	9.57×10^{-7}
Hypertension	-0.130	0.878	0.748	1.031	-1.584	1.13×10^{-1}
Diabetes mellitus	0.078	1.081	0.910	1.285	0.885	3.76×10^{-1}
Dyslipidemia	0.047	1.048	0.894	1.229	0.582	5.61×10^{-1}
Smoking history	-0.044	0.957	0.817	1.120	-0.548	5.84×10^{-1}
LVEF (%)	-0.004	0.996	0.991	1.002	-1.403	1.61×10^{-1}
Log_{10} (NT-proBNP) (pg/ml)	0.509	1.664	1.406	1.971	5.908	3.47×10^{-9}
Hb (g/dl)	-0.157	0.855	0.824	0.887	-8.417	3.87×10^{-17}
eGFR ($ml\cdot min^{-1}\cdot 1.73\ m^{-2}$)	-0.019	0.982	0.978	0.986	-8.977	2.78×10^{-19}
X-ray Age (years)	0.040	1.040	1.031	1.050	8.314	9.29×10^{-17}

Coefficients of the univariate Cox proportional hazards model for the primary endpoint of HF patients. Hypertension, diabetes mellitus, dyslipidemia, and smoking history were treated as binary categorical variables. HR, hazard ratio; BMI, body mass index; LVEF, left ventricular ejection fraction; Hb, hemoglobin; eGFR, estimated glomerular filtration rate

Supplementary Table 10. Comparison of different Cox proportional hazards models and improved predictive performance due to addition of age discrepancy to models

Model	Covariates in Cox model	AIC	Performance improvement												
			Compared with	IDI				cNRI				MI			
				Estimate	lower 95%	upper 95%	P value	Estimate	lower 95%	upper 95%	P value	Estimate	lower 95%	upper 95%	P value
Model 1	Age + Sex + BMI + LVEF +log(NT-proBNP) + Hb + eGFR	7608.6													
Model 2	Age + Sex + BMI + LVEF +log(NT-proBNP) + Hb + eGFR + Age_discrepancy	7206.6	vs Model 1	0.0104	0.0007	0.0245	0.020	0.134	0.025	0.201	0.010	0.0110	0.0008	0.0250	0.020
Model 3	Age + Sex + BMI + LVEF +log(NT-proBNP) + Hb + eGFR + CXR_Abnormality	7204.4													
Model 4	Age + Sex + BMI + LVEF +log(NT-proBNP) + Hb + eGFR + CXR_Abnormality + Age_discrepancy	7198.9	vs Model 3	0.0105	0.0007	0.0251	0.030	0.135	0.034	0.202	0.030	0.0110	0.0008	0.0260	0.030
Model 5	Age + Sex + BMI + LVEF +log(NT-proBNP) + Hb + eGFR + CXR_Abnormality_score	7206.1													
Model 6	Age + Sex + BMI + LVEF +log(NT-proBNP) + Hb + eGFR + CXR_Abnormality_score + Age_discrepancy	7200.4	vs Model 5	0.0105	0.0008	0.0250	0.020	0.121	0.036	0.197	0.020	0.0120	0.0008	0.0250	0.020

Comparison of different Cox proportional hazards models with different variables and the additional value of age discrepancy as assessed by the paired difference of risk scores derived from the Cox proportional hazard model is shown. Improvement in

the predictive performance of the Cox model assessed by Akaike's Information Criterion (AIC), integrated discrimination improvement (IDI); continuous net reclassification improvement (cNRI), and median improvement (MI).

BMI, body mass index; LVEF, left ventricular ejection fraction; Hb, hemoglobin; eGFR, estimated glomerular filtration rate; X-ray age, age estimated using the deep learning model; Age_discrepancy, the difference between X-ray age and chronological age (X-ray age - chronological age); CXR_abnormality, binary variable for whether CXR is abnormal or not from abnormality detection deep learning model; CXR_abnormality_score, continuous variable indicating whether there are abnormalities in CXR from abnormality detection deep learning model.

Supplementary Table 11. Characteristics of patients in the MIMIC database

**clinical measurements (unit)**	**(n=3586)**
Age (years old)	71 [61, 80]
The number of males (%)	2097 (58.5)
Height (cm)	170 [163, 178]
Weight (kg)	80.6 [67.7, 95.95]
BMI (kg/m2)	28.1 [24.5, 32.8]
Medical history	
myocardial_infarction	1666 (46.5)
congestive_heart_failure	2788 (77.7)
peripheral_vascular_disease	624 (17.4)
chronic_pulmonary_disease	1320 (36.8)
cerebrovascular_disease	384 (10.7)
diabetes	1480 (41.3)
Systolic BP (mmHg)	114 [105, 125]
Diastolic BP (mmHg)	61 [54, 68]
Heart rate (/min)	83 [73, 95]
SpO2 (%)	97 [95, 98]
Respiratory rate (/min)	20 [17, 22]
Laboratory measurements	
Hb (g/dl)	11.4 [9.8, 13.0]
Hct (%)	35.0 [30.6, 39.9]
WBC (10^3)	12.60 [9.0, 17.4]
PLT (10^3)	209 [158, 273]
Na (mEq/L)	139 [137, 142]
K (mEq/L)	4.5 [4.11, 5.0]
T-Bil (mg/dl)	0.7 [0.4, 1.2]
AST (U/L)	45 [26, 105]
ALT (U/L)	30 [18, 66]
ALP (U/L)	87 [65, 124]
LDH (U/L)	302 [218, 494.5]
Alb (mg/dl)	3.4 [2.9, 3.8]
CRP (mg/L)	71.30 [24.57, 147.55]
Cre (mg/dl)	1.30 [1.00, 2.10]
eGFR (ml·min-1·1.86 m-2)	51.33 [29.69, 74.73]
Glucose (mg/dl)	134 [115, 169]
TP (mg/dl)	6.0 [5.4, 6.4]

Characteristics of cardiovascular disease patients in MIMIC database. Continuous variables are presented as medians [interquartile ranges]. Categorical variables are presented as n (%). BMI, body mass index; BP, blood pressure; Hb, hemoglobin; Hct, hematocrit; WBC, white blood cell count; PLT, platelet count; T-Bil, total bilirubin; AST, aspartate aminotransferase; ALT, alanine aminotransferase; ALP, alkaline phosphatase; UA, urinary acid; CRP, C-reactive protein; Cre, creatinine; eGFR, estimated glomerular filtration rate; Lymph, lymphocytes; BS, blood glucose; TP, total protein.

Supplementary Table 12. Multivariate Cox proportional hazards model in MIMIC data.

	Coefficient	HR	C.I.		Z score	P value
			lower 95%	upper 95%		
Age (years)	0.0261	1.0264	1.0175	1.0354	5.8507	4.9×10^{-9}
Sex (Male)	0.2003	1.2218	1.0268	1.4538	2.2579	2.4×10^{-2}
eGFR (ml·min-1·1.73 m-2)	-0.0104	0.9896	0.9865	0.9927	-6.5361	6.3×10^{-11}
Hb (g/dl)	-0.0504	0.9509	0.9136	0.9897	-2.4689	1.4×10^{-2}
CHF	-0.1334	0.8751	0.6812	1.1243	-1.0434	3.0×10^{-1}
MI	0.0227	1.0230	0.8381	1.2487	0.2234	8.2×10^{-1}
Age discrepancy (years)	0.0163	1.0165	1.0027	1.0305	2.3403	1.9×10^{-2}

Multivariate Cox proportional hazards analysis. The P value was calculated using the 100,000 bootstrap replications method. BMI, body mass index; LVEF, left ventricular ejection fraction; Hb, hemoglobin; eGFR, estimated glomerular filtration rate; CHF, congestive heart failure; MI, myocardial infarction; Age discrepancy, difference between X-ray age and chronological age (X-ray age - chronological age).

Supplementary Figures

Supplementary Fig. 1 Age and sex distribution in the datasets.

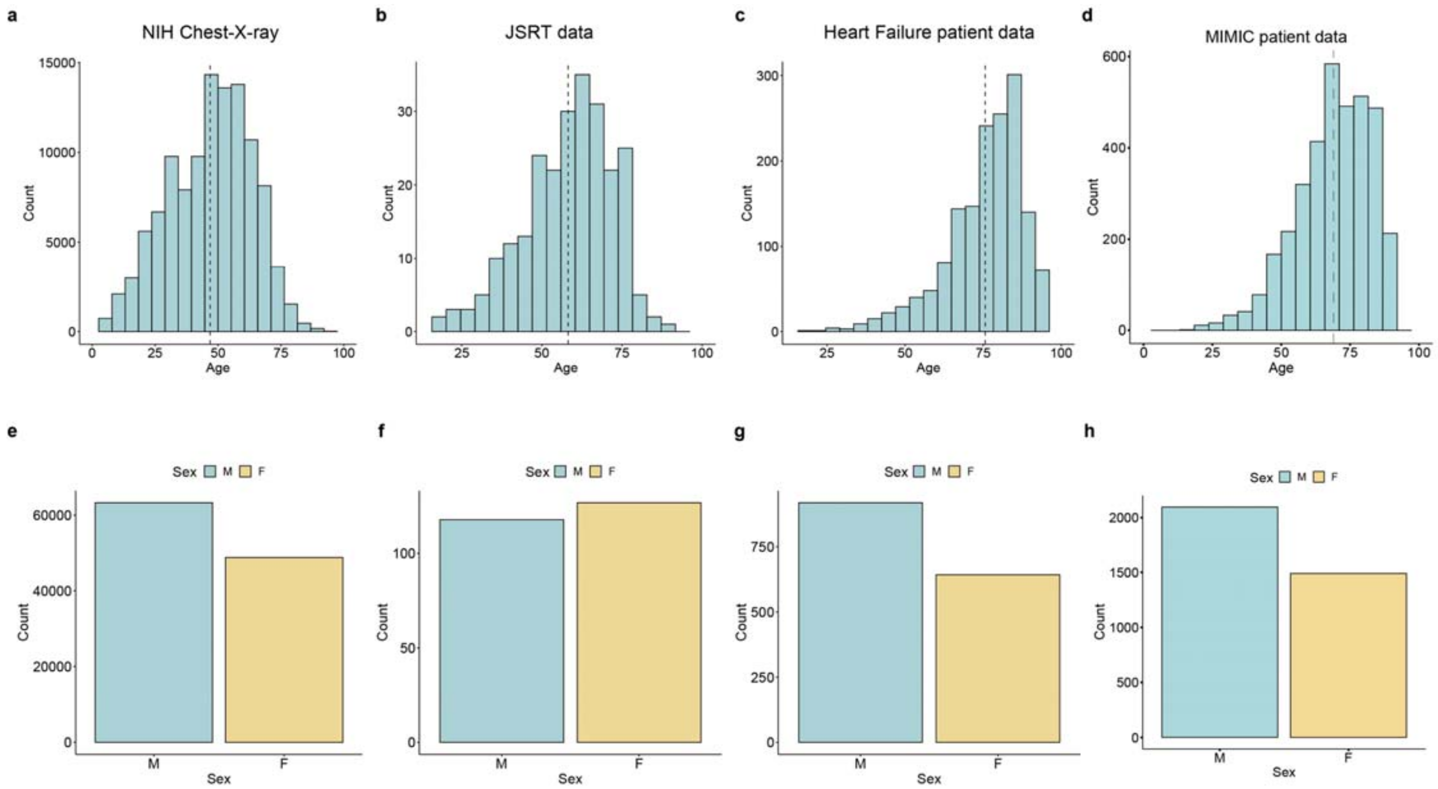

Histogram of age and sex in the NIH Chest X-ray database (**a**, **e**), JSRT database (**b**, **f**), heart failure patient data (**c**, **g**), and MIMIC data (d, h). M, male; F, female.

Supplementary Fig. 2 Study flowchart and data usage.

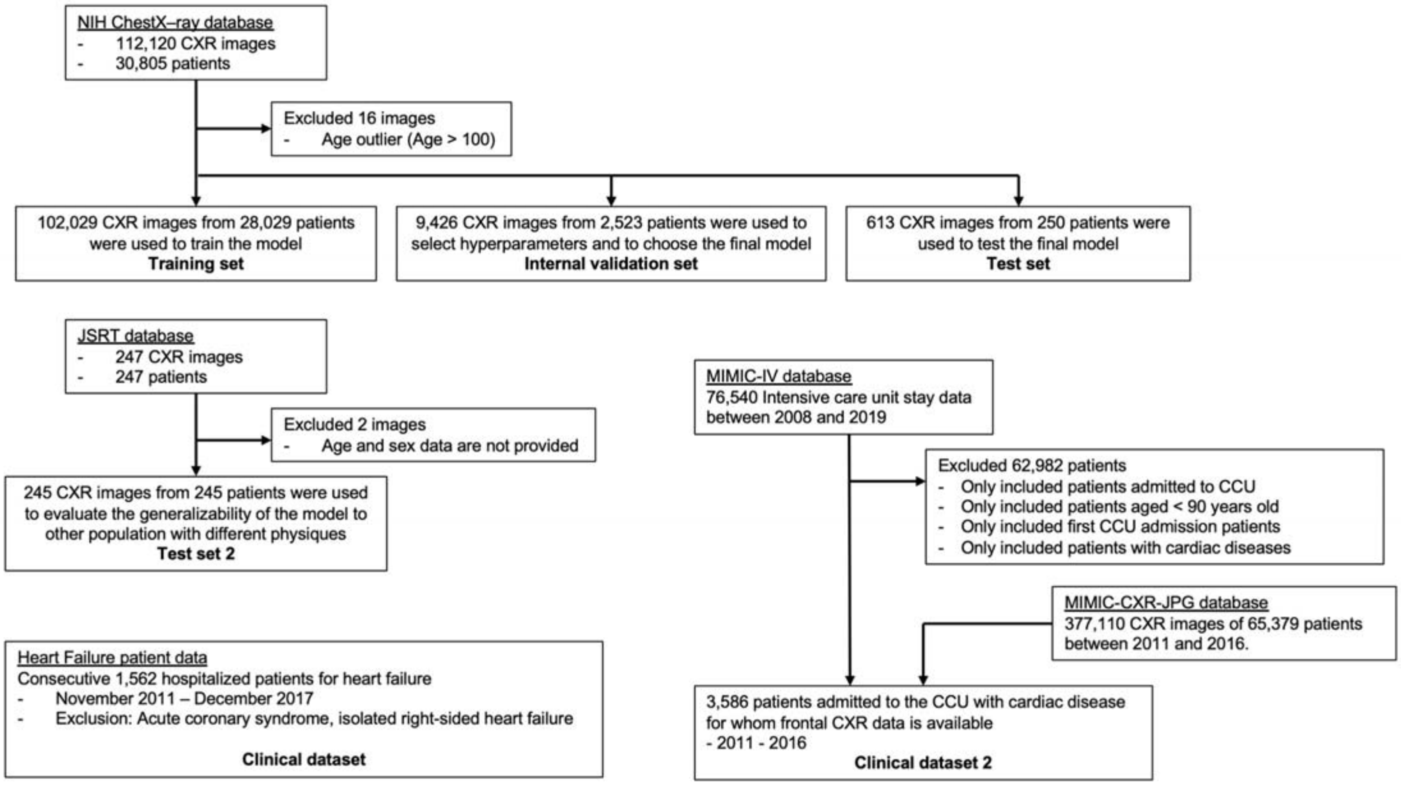

Four datasets were used in this study. The NIH Chest X-ray database was randomly divided into training, validation, and test datasets after excluding 16 age outliers. The JSRT database was used as independent external test data to evaluate the predictive performance of the deep learning model. Two patients whose age and sex data were unavailable were excluded. The heart failure patient data consisted of 1,562 consecutive patients who were admitted to the Sakakibara Heart Institute. From the MIMIC-IV and MIMIC-CXR databases, 3,586 patients admitted to the CCU with cardiac disease were identified and their data were extracted. These data were used to explore the clinical significance of the estimated age in real-world patient data.

Supplementary Fig. 3 Estimation accuracy of the deep learning model in validation data.

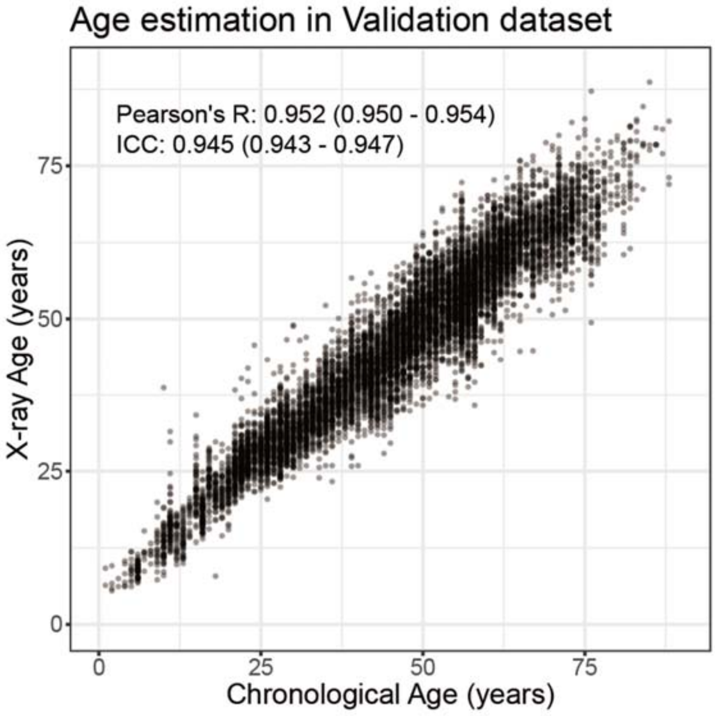

Scatter plot of the chronological age (x-axis) and estimated age (y-axis) with Pearson's correlation coefficient and intraclass correlation coefficient case1 (ICC) in the validation dataset.

Supplementary Fig. 4 Reproducibility analysis.

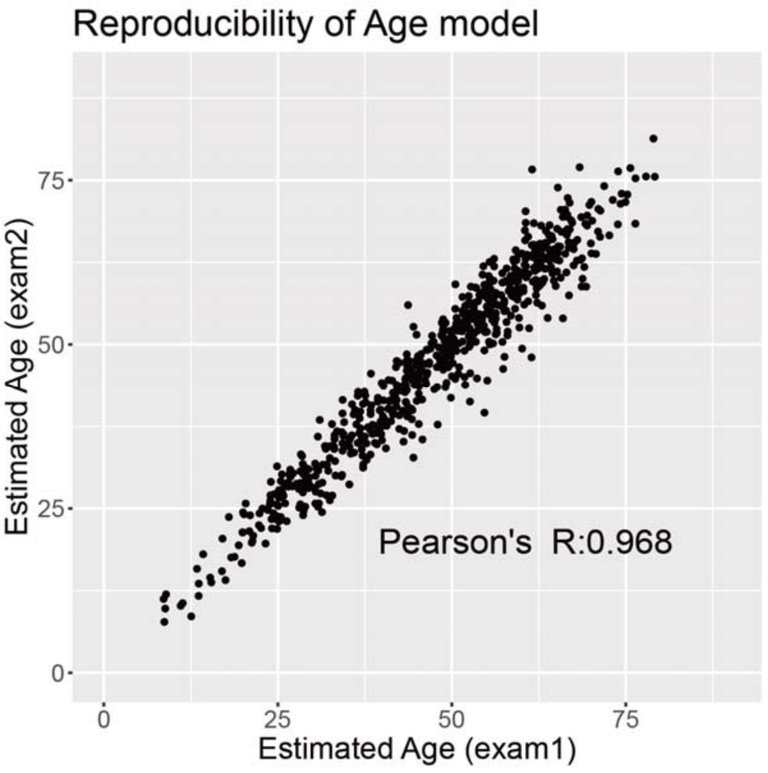

Scatter plot of the estimated age of different CXRs from the same patient. The estimated ages of the first and second exams are plotted on the x- and y-axes, respectively

Supplementary Fig. 5 Comparison of age estimation performance between human experts and DNN model

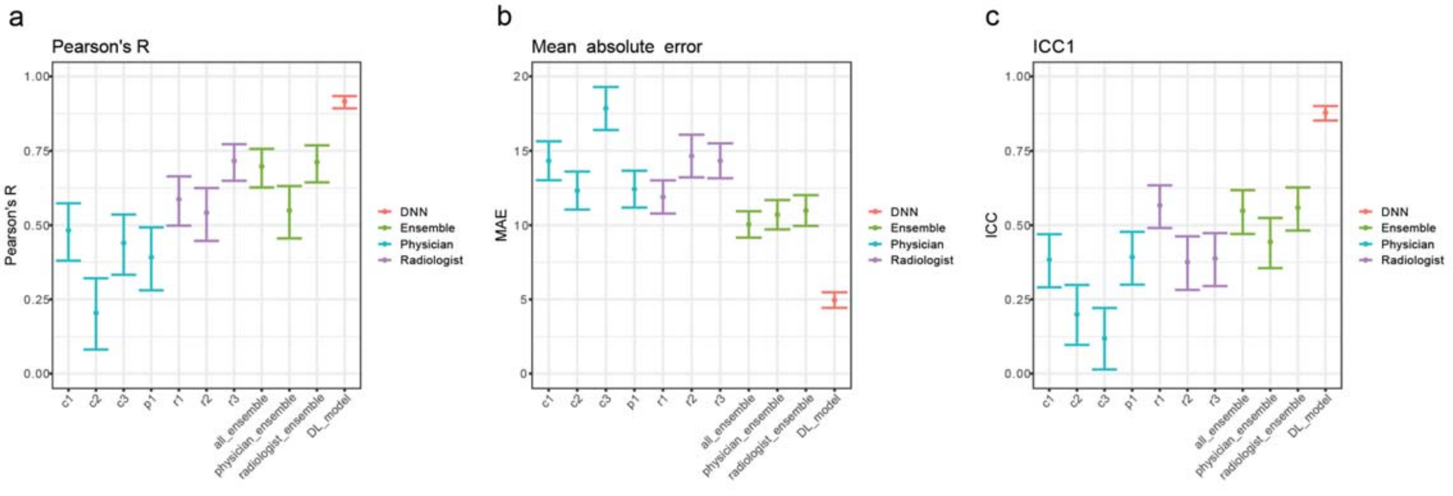

Different metrics of age estimation performance in the JSRT dataset are shown. (a) Pearson's R, (b) Mean absolute error, (c) Intraclass correlation coefficient between estimated and chronological age. c1-c3, cardiologist; p1, pulmonologist; r1-r3, radiologist; MAE, mean absolute error; ICC, intraclass correlation coefficient; DL_model, deep learning model

Supplementary Fig. 6 Visualization of CXR images with each finding label using Grad-CAM and Guided-back propagation

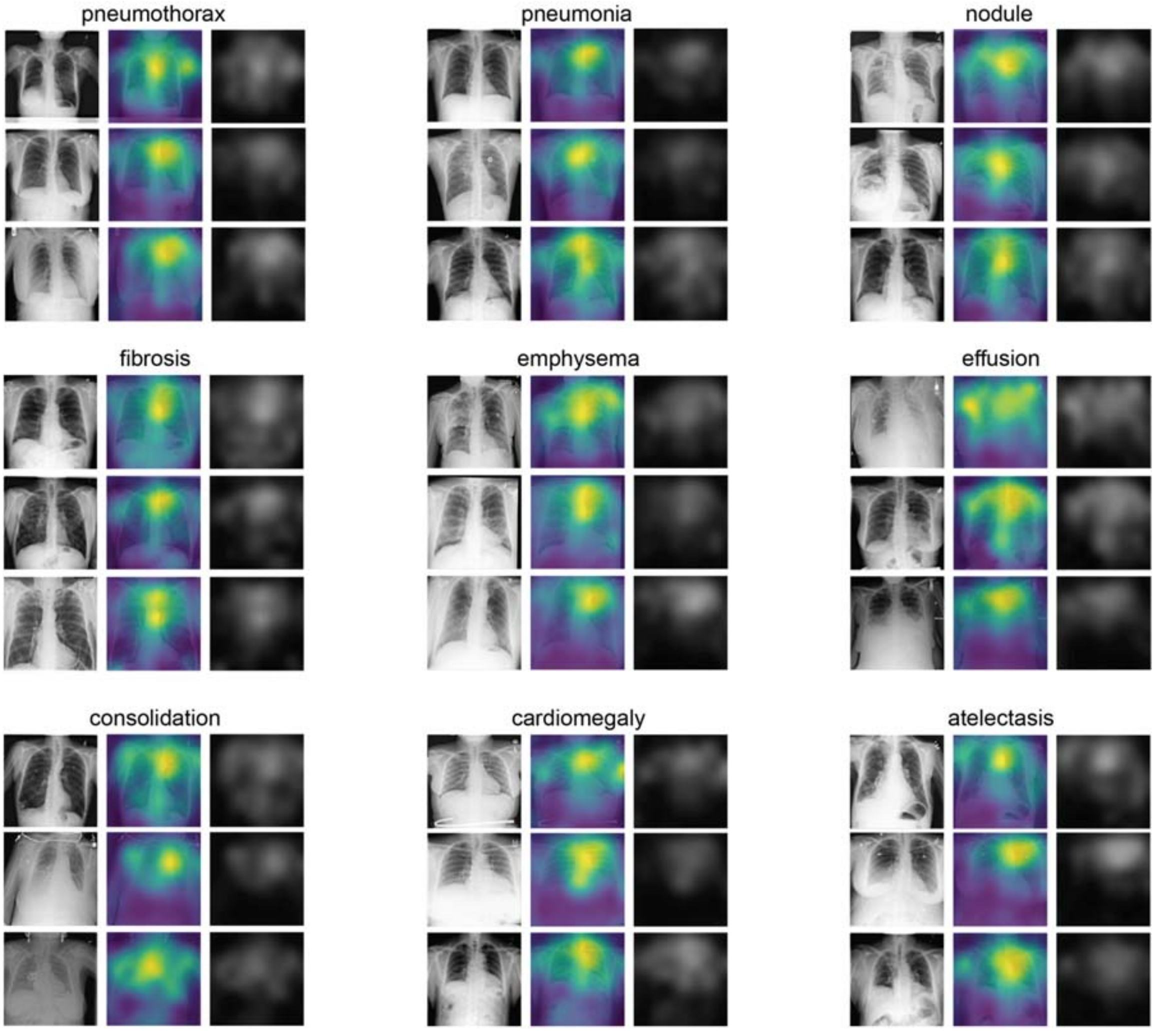

Example of CXRs with finding labels are shown on left side with the finding labels on top. Heatmap visualization using Grad-CAM (middle) and guided Grad-CAM (right) for each of the CXRs are shown.

Supplementary Fig. 7 Adjusted survival rate stratified by the age discrepancy in MIMIC data.

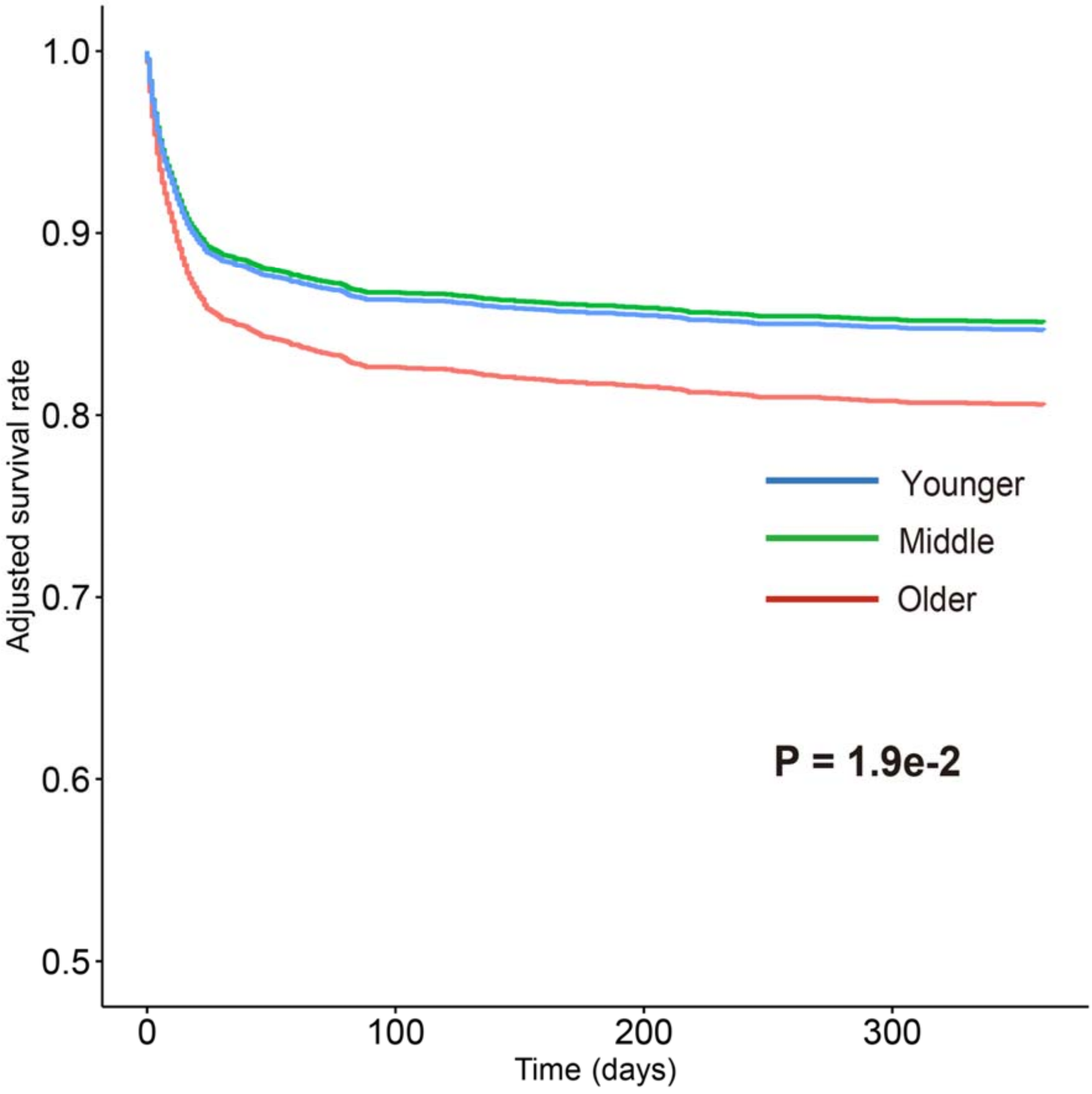

Adjusted survival curve for patients in MIMIC data stratified by age discrepancy between the chronological age and X-ray age. Event was defined as all-cause mortality. The top 20% of patients, middle 60%, and bottom 20% were grouped as older, middle, and younger, respectively.

Supplementary Fig. 8 Difference in prognostic impact of X-ray age in younger and elderly patients.

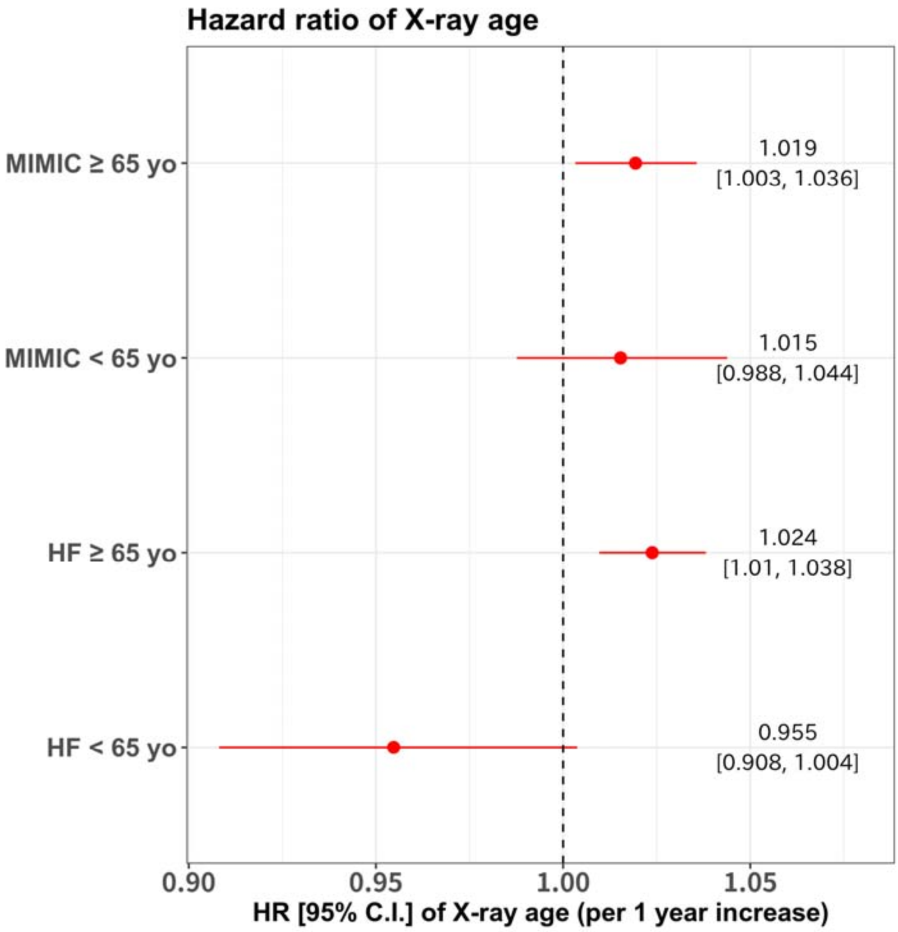

The hazard ratio of the age discrepancy per 1 year increase in X-ray age derived by multivariate Cox proportional hazard model in the MIMIC database and heart failure cohort data is given. Age discrepancy was a significant prognostic marker in older patients (≥ 65 years old), but not in younger patients (< 65 years old), both in the MIMIC database and heart failure (HF) cohort. HF, heart failure; HR, hazard ratio; C.I., confidence interval
