# Supplementary material for: Deep learning-based age estimation from chest X-rays indicates cardiovascular prognosis
Source: Commun Med (Lond). 2022 Dec 9;2:159. doi: 10.1038/s43856-022-00220-6 (PMC9734197; doi:10.1038/s43856-022-00220-6)
Supplement: Supplementary file 9 — Description of Additional Supplementary Files [file 43856_2022_220_MOESM9_ESM.pdf]

Description of Additional Supplementary Files

File Name: Supplementary Data 1

Description: X-ray Age and chronological age in the test dataset

File Name: Supplementary Data 2

Description: X-ray Age and chronological age in the JSRT dataset

File Name: Supplementary Data 3

Description: Physicians' estimated age and chronological age in the JSRT dataset

File Name: Supplementary Data 4

Description: Relationship between age estimation error and presence of any finding labels
OR, odds ratio; CI, confidence interval

File Name: Supplementary Data 5

Description: Different finding labels that affect the patient's estimated age
OR, odds ratio; CI, confidence interval

File Name: Supplementary Data 6

Description: Relationship between patients' clinical history and X-ray age
OR, odds ratio; CI, confidence interval; HTN, hypertension; DM, diabetes mellitus; DL, dyslipidemia; HUA, hyperuricemia; AFAFL, atrial fibrillation or atrial flutter; COPD, chronic obstructive pulmonary disease; device, cardiac pacemaker, implantable cardioverter defibrillator, or cardiac resynchronization therapy devices

File Name: Supplementary Data 7

Description: Relationship between patients' clinical measurements and X-ray age
OR, odds ratio; CI, confidence interval; LAD, left atrial diameter; LVEF, left ventricular ejection fraction; LVDd, left ventricular end-diastolic diameter; TC, total cholesterol; BS, blood sugar (glucose); HR, heart rate; dBP, diastolic blood pressure; sBP, systolic blood pressure
