# Supplementary material for: Deep learning-based age estimation from chest X-rays indicates cardiovascular prognosis
Source: Commun Med (Lond). 2022 Dec 9;2:159. doi: 10.1038/s43856-022-00220-6 (PMC9734197; doi:10.1038/s43856-022-00220-6)
Supplement: Supplementary file 10 — Reporting Summary [file 43856_2022_220_MOESM10_ESM.pdf]

nature portfolio

Corresponding author(s): Kaoru Ito

Last updated by author(s): Nov 21, 2022

Reporting Summary

Nature Portfolio wishes to improve the reproducibility of the work that we publish. This form provides structure for consistency and transparency in reporting. For further information on Nature Portfolio policies, see our Editorial Policies and the Editorial Policy Checklist.

Statistics

For all statistical analyses, confirm that the following items are present in the figure legend, table legend, main text, or Methods section.

n/a	Confirmed	
☐	☒	The exact sample size (n) for each experimental group/condition, given as a discrete number and unit of measurement
☐	☒	A statement on whether measurements were taken from distinct samples or whether the same sample was measured repeatedly
☐	☒	The statistical test(s) used AND whether they are one- or two-sided<br>*Only common tests should be described solely by name; describe more complex techniques in the Methods section.*
☐	☒	A description of all covariates tested
☐	☒	A description of any assumptions or corrections, such as tests of normality and adjustment for multiple comparisons
☐	☒	A full description of the statistical parameters including central tendency (e.g. means) or other basic estimates (e.g. regression coefficient) AND variation (e.g. standard deviation) or associated estimates of uncertainty (e.g. confidence intervals)
☐	☒	For null hypothesis testing, the test statistic (e.g. F, t, r) with confidence intervals, effect sizes, degrees of freedom and P value noted<br>*Give P values as exact values whenever suitable.*
☒	☐	For Bayesian analysis, information on the choice of priors and Markov chain Monte Carlo settings
☒	☐	For hierarchical and complex designs, identification of the appropriate level for tests and full reporting of outcomes
☐	☒	Estimates of effect sizes (e.g. Cohen's d, Pearson's r), indicating how they were calculated

Our web collection on statistics for biologists contains articles on many of the points above.

Software and code

Policy information about availability of computer code

Data collection	No software was used to collect data from Sakakibara heart failure patient cohorts.<br><br>Besides, we used publically available data as listed below.<br>NIH Chest X-ray database, https://cloud.google.com/healthcare/docs/resources/public-datasets/nih-chest;<br>JSRT database, http://db.jsrt.or.jp/eng.php<br>MIMIC-IV database, https://physionet.org/content/mimiciv/1.0/<br>MIMIC-CXR-JPG, https://physionet.org/content/mimic-cxr-jpg/2.0.0/<br>Further details are described in the Method section.
Data analysis	We used open source softwares for the analysis, as listed below.<br>R(4.1.0), https://www.r-project.org;<br>boot(1.3.28), https://cran.r-project.org/src/contrib/Archive/boot/;<br>tidyverse(1.3.1), https://www.tidyverse.org;<br>MASS(7.3.55), https://cran.r-project.org/src/contrib/Archive/MASS/;<br>survival(3.3.1), https://cran.r-project.org/src/contrib/Archive/survival/;<br>survminer(0.4.9), https://cran.r-project.org/web/packages/survminer/;<br>survIDINRI(1.1.1), https://cran.r-project.org/web/packages/survIDINRI/;<br>psych(2.1.9), https://cran.r-project.org/web/packages/psych/;<br>Python(3.8.12), https://www.python.org/downloads/release/python-376/;<br>PyTorch(1.4.0), https://pytorch.org/get-started/previous-versions/;<br>Matplotlib(3.2.2), https://github.com/matplotlib/matplotlib;<br>numpy(1.22.3), https://github.com/numpy/numpy;<br>fastai(1.0.61), https://github.com/fastai/fastai1;<br>opencv(4.5.5), https://github.com/opencv/opencv-python;

pydicom(1.4.2), https://github.com/pydicom/pydicom;
pandas(1.4.1), https://github.com/pandas-dev/pandas;
scikit-learn(0.23.1), https://github.com/scikit-learn/scikit-learn;
pretrainedmodels(0.7.4), https://github.com/Cadene/pretrained-models.pytorch;
xray_age(0.1.0), https://github.com/pirocv/xray_age

Further details are described in the Method section.

For manuscripts utilizing custom algorithms or software that are central to the research but not yet described in published literature, software must be made available to editors and reviewers. We strongly encourage code deposition in a community repository (e.g. GitHub). See the Nature Portfolio guidelines for submitting code & software for further information.

Data

Policy information about availability of data

All manuscripts must include a data availability statement. This statement should provide the following information, where applicable:

- Accession codes, unique identifiers, or web links for publicly available datasets
- A description of any restrictions on data availability
- For clinical datasets or third party data, please ensure that the statement adheres to our policy

The data generated and analyzed during this study are available from the corresponding authors upon request. The NIH chest X-ray dataset used in this study is openly available and can be downloaded at https://cloud.google.com/healthcare/docs/resources/public-datasets/nih-chest. The JSRT database used in this study is publicly available and can be downloaded at http://db.jsrt.or.jp/eng.php. Heart failure patients' data is available upon reasonable request. The MIMIC-IV and MIMIC-CXR-JPG databases used in this study are publicly available and can be downloaded at https://physionet.org/content/mimiciv/1.0/ and https://physionet.org/content/mimic-cxr-jpg/2.0.0/, respectively. Source data for the Figures 2, 4, and 5 are available as Supplementary Data 1-3, 4-5, 6-7, respectively.

Field-specific reporting

Please select the one below that is the best fit for your research. If you are not sure, read the appropriate sections before making your selection.

☒ Life sciences ☐ Behavioural & social sciences ☐ Ecological, evolutionary & environmental sciences

For a reference copy of the document with all sections, see nature.com/documents/nr-reporting-summary-flat.pdf

Life sciences study design

All studies must disclose on these points even when the disclosure is negative.

Sample size	Because we aimed to create the largest sample size in each dataset / cohort in order to increase the performance of a classifier / gain the statistical power, we included as many case and control individuals as possible in our analysis.
Data exclusions	We excluded individuals according to the standard quality control procedure. Further details are described in the Method section.
Replication	In order to develop our deep learning algorithm, we divided the NIH dataset into training, validation and test datasets. Subsequently, we checked the performance using the JSRT dataset. For survival analysis, we used samples from the Sakakibara heart failure patient cohort and patients with cardiovascular diseases from MIMIC-IV database.
Randomization	Randomization is not applicable because of the study purpose.
Blinding	Blinding is not applicable because of the study purpose.

Reporting for specific materials, systems and methods

We require information from authors about some types of materials, experimental systems and methods used in many studies. Here, indicate whether each material, system or method listed is relevant to your study. If you are not sure if a list item applies to your research, read the appropriate section before selecting a response.

Materials & experimental systems

n/a	Involved in the study	
☒	☐	Antibodies
☒	☐	Eukaryotic cell lines
☒	☐	Palaeontology and archaeology
☒	☐	Animals and other organisms
☐	☒	Human research participants
☒	☐	Clinical data
☒	☐	Dual use research of concern

Methods

n/a	Involved in the study	
☒	☐	ChIP-seq
☒	☐	Flow cytometry
☒	☐	MRI-based neuroimaging

Human research participants

Policy information about studies involving human research participants

Population characteristics	1. NIH Chest X-ray database<br>NIH Chest X-ray database is a hospital-scale database of CXRs and NLP-processed finding labels collected at NIH clinical center.<br><br>2. JSRT database<br>JSRT database is a CXR database of Japanese patients which is collected from 13 medical centers in Japan.<br><br>3. Sakakibara heart failure patient cohort<br>Sakakibara heart failure patient cohort is a hospital-based prospective registry, which enrolls acute decompensated heart failure patients.<br><br>4. MIMIC database<br>MIMIC database is a large publicly-available database comprising of deidentified health-related data from patients who were admitted to the critical care units of the Beth Israel Deaconess Medical Center.
Recruitment	1. Sakakibara heart failure patient cohort<br>Acute decompensated heart failure patients who admitted to Sakakibara Heart Institute between 2011 and 2017 were recruited. This database collects conventional clinical variables (including vital signs, laboratory findings, treatment during hospitalization and medical imaging test results) and patient outcome (re-hospitalization and survival) information.
Ethics oversight	1. Sakakibara heart failure patient cohort<br>Study protocol was approved by the institutional review Board of the Sakakibara Heart Institute (No. 19-092). Written informed consent were obtained from all participants.

Note that full information on the approval of the study protocol must also be provided in the manuscript.
